# Syntenic relationships between cucumber (*Cucumis sativus *L.) and melon (*C. melo *L.) chromosomes as revealed by comparative genetic mapping

**DOI:** 10.1186/1471-2164-12-396

**Published:** 2011-08-05

**Authors:** Dawei Li, Hugo E Cuevas, Luming Yang, Yuhong Li, Jordi Garcia-Mas, Juan Zalapa, Jack E Staub, Feishi Luan, Umesh Reddy, Xiaoming He, Zhenhui Gong, Yiqun Weng

**Affiliations:** 1 Horticulture College, Northwest A & F University, Yangling 712100, China; 2 Horticulture Department, University of Wisconsin, Madison, WI 53706, USA; 3 USDA ARS Tropical Agriculture Research Station, Mayaguez, P.R. 00680, Puerto Rico; 4 IRTA, Center for Research in Agricultural Genomics CSIC-IRTA-UAB, Campus UAB, Edifici CRAG, 08193 Bellaterra (Barcelona), Spain; 5 USDA ARS Vegetable Crops Research Unit, Horticulture Department, University of Wisconsin, Madison, WI 53706, USA; 6 USDA-ARS, Forage & Range Research Laboratory, Utah State University, Logan, UT 84322 USA; 7 Horticulture College, Northeast Agricultural University, Harbin, 150030, China; 8 Department of Biology, West Virginia State University Institute, WV 25112, USA; 9 Vegetable Research Institute, Guangdong Academy of Agricultural Sciences, Guangzhou 510640, China

**Keywords:** Cucumber, Melon, *Cucumis*, Microsatellite, Comparative mapping, Chromosome evolution

## Abstract

**Background:**

Cucumber, *Cucumis sativus *L. (2n = 2 × = 14) and melon, *C. melo *L. (2n = 2 × = 24) are two important vegetable species in the genus *Cucumis *(family Cucurbitaceae). Both species have an Asian origin that diverged approximately nine million years ago. Cucumber is believed to have evolved from melon through chromosome fusion, but the details of this process are largely unknown. In this study, comparative genetic mapping between cucumber and melon was conducted to examine syntenic relationships of their chromosomes.

**Results:**

Using two melon mapping populations, 154 and 127 cucumber SSR markers were added onto previously reported F_2_- and RIL-based genetic maps, respectively. A consensus melon linkage map was developed through map integration, which contained 401 co-dominant markers in 12 linkage groups including 199 markers derived from the cucumber genome. Syntenic relationships between melon and cucumber chromosomes were inferred based on associations between markers on the consensus melon map and cucumber draft genome scaffolds. It was determined that cucumber Chromosome 7 was syntenic to melon Chromosome I. Cucumber Chromosomes 2 and 6 each contained genomic regions that were syntenic with melon chromosomes III+V+XI and III+VIII+XI, respectively. Likewise, cucumber Chromosomes 1, 3, 4, and 5 each was syntenic with genomic regions of two melon chromosomes previously designated as II+XII, IV+VI, VII+VIII, and IX+X, respectively. However, the marker orders in several syntenic blocks on these consensus linkage maps were not co-linear suggesting that more complicated structural changes beyond simple chromosome fusion events have occurred during the evolution of cucumber.

**Conclusions:**

Comparative mapping conducted herein supported the hypothesis that cucumber chromosomes may be the result of chromosome fusion from a 24-chromosome progenitor species. Except for a possible inversion, cucumber Chromosome 7 has largely remained intact in the past nine million years since its divergence from melon. Meanwhile, many structural changes may have occurred during the evolution of the remaining six cucumber chromosomes. Further characterization of the genomic nature of *Cucumis *species closely related to cucumber and melon might provide a better understanding of the evolutionary history leading to modern cucumber.

## Background

The genus *Cucumis *(family Cucurbitaceae) includes two economically important vegetable crop species that are cultivated worldwide: cucumber (*C. sativus *L., 2n = 2 × = 14) and melon (*C. melo *L., 2n = 2 × = 24). The genetic, phylogenetic, and evolutionary relationships of cucumber and melon have been the subject of much research. The genus *Cucumis *initially contained 32 species that was divided into two subgenera, *Melo *and *Cucumis *[1]. While the subgenus *Melo *is centered in Africa with 30 species including melon (all of which have 2n = 24 chromosomes), the subgenus *Cucumis *is of Asian origin and includes the cultivated cucumber *C. sativus *and its wild relative *C. hystrix *Char. (2n = 2 × = 24). Although *C. melo *is considered the most morphologically diverse species in *Cucumis *[1,2], two inter-fertile botanical varieties (2n = 2 × = 14), the cultivated *C. sativus *var. *sativus *L. and the wild *C. sativus *var. *hardwickii *(Royle) Alef., comprise the primary gene pool of cucumber. This gene pool has a rather narrow genetic base as evidenced in various genetic diversity studies [3-6]. No interspecific hybrids between melon and cucumber have been reported due to their sexual incompatibility [7].

The genus *Cucumis *has undergone considerable revision in recent years. For instance, molecular phylogenetic studies indicated that the genera such as *Cucumella*, *Mukia*, *Dicaelospermum*, *Myrmecosicyos*, and *Oreosyce *possess genetic affinities with *Cucumis *species resulting in their inclusion in *Cucumis *in more recent taxonomic treatments [8-10]. The genus *Cucumis *in the newest treatment contains 52 species, which are grouped into two subgenera: *Humifructus *(two species, *C. humifructus *and *C. hirsutus*) and *Cucumis *(consisting of the remaining 50 species) [9,11]. In addition, both melon and cucumber are believed to be of Asian origin, which were derived from a common ancestor approximately nine million years ago [12].

The genome size of melon (12 chromosome pairs) is estimated to be 454 Mb, and cucumber (7 chromosome pairs) has a genome size of 367 Mbp [13]. The evolutionary relationship between melon and cucumber can be investigated through chromosome analysis. In Kirkbride's taxonomic assessment of *Cucumis *[2], subgenus *Cucumis *is considered primitive and subgenus *Melo *was hypothesized to have been derived from it through chromosomal fragmentation [14-16]. In contrast, cytological investigations have also suggested that ancestral species of subgenus *Melo *gave rise to subgenus *Cucumis *species via chromosome fusion or non-homologous translocation [17,18]. However, Ramachandran and Seshadri [19] argued that the two subgenera are not closely related given differences in geographical distribution and chromosome number, size, organization, and behavior. More recent molecular-based phylogenetic analyses of *Cucumis *support the hypothesis that the base chromosome number of *x *= 7 was achieved by chromosome reduction from *x *= 12 progenitor species [8,10,12].

Despite their distinct phylogenetic relationships [20,21], the genomes of melon and cucumber seem to be highly conserved. Cross-species similarities based on molecular marker transferability among cucurbit crops are well documented. Neuhausen [20] first reported affinities among cucurbit species by identifying molecular cross-hybridizations (i.e., signals) using RFLP probes. More recently, Katzir et al. [21] and Danin-Poleg et al. [22] defined specific genomic regions using SSR primer products to reveal considerable sequence homologies between cucumber and melon. Danin-Poleg et al. [23] identified nine SSR markers shared between melon and cucumber and proposed that their cucumber Linkage Group B and melon Linkage Groups E and 2 were syntenic. Since 2000, molecular markers (primarily SSRs) developed from melon have been used routinely in cucumber genetic mapping studies or vice versa [24-37]. The high degree of synteny and conservation between the melon and cucumber genomes has also been demonstrated at the DNA sequence level (micro-synteny). Park et al. [38] and Meyer et al. [39] compared genomic DNA flanking the zucchini yellow mosaic virus resistance locus (*zym*) in melon and cucumber and detected considerable marker colinearity between those species. Alignment of melon BAC-end and BAC clone (6.7 Mbp) sequences of melon against a cucumber draft genome assembly (line Gy 14) revealed 90% homology between the compared sequences [40,41]. Although transposition activity was found to be low in cucumber, it is comparatively high in melon [41]. Thus, it has been postulated that the genomic size differences between melon and cucumber is due mainly to the expansion of inter-genic regions and proliferation of transposable elements in the melon genome [41].

Recently, whole genome sequencing in cucumber [42] and the availability of large numbers of molecular markers [43] has made it possible to define more clearly the syntenic relationships between cucumber and melon. By alignment of 348 marker sequences mapped in the melon genome onto the 9930 cucumber draft genome, Huang et al. [42] found that there was no substantial rearrangement between cucumber Chromosome 7 and melon Chromosome I. In addition, the majority of cucumber Chromosome 4 corresponded to melon Chromosome VII, and each of the remaining five cucumber chromosomes was collinear to two melon chromosomes [42]. The correspondance between melon and cucumber chromosomes was also observed in a comparative mapping study by Fukino et al. [44], who placed 70 cucumber SSR markers on a melon linkage map.

Comparative genetic mapping is useful for revealing syntenic relationships among closely related planted species [45,46]. An understanding of syntenic relationships among species facilitates the investigation of genome evolution and dynamics [47,48], and allows for the use of genetic information among related species in gene isolation and molecular tagging experiments [49-51]. Comparative mapping has been used successfully to define syntenic relationships among closely related plant species in the Solanaceae (pepper, tomato, and potato) [52-55], Gramineae grasses [56,57], Fabaceae legumes [47,58-62], Brassicaceae [63], and Rosaceae (*Prunun *spp) [64,65].

When compared to other crop species, genetic and genomic resources in cucurbit crops have historically been limited. However, this situation is changing rapidly. For instance, the draft genomes of two cucumber inbred lines (North China fresh market type 9930 and North American pickling type Gy14) have been released [42,66] (also Weng et al., unpublished data). A high resolution linkage map and several SSR-based genetic maps have been developed for this species [43,67-69]. In melon, many linkage maps as well as a BAC-based physical map have been constructed [24,27-29,33-37]. In addition, comparative fluorescence *in situ *hybridization (FISH) mapping in cucumber and melon [70] suggested that centromere repositioning occurred during the evolution of cucumber chromosomes. Several cucumber chromosomes have been anchored using fosmid clones, and karyotypes of cucumber and melon genomes have been developed [71-75].

Previous studies [38-44] have shown that genomic synteny and co-linearity exists between cucumber and melon. This information is, nevertheless, fragmented and incomplete. Thus experiments were designed herein for large-scale comparative genetic mapping of melon and cucumber to identify syntenic blocks using microsatellite markers. To accomplish this objective, two extended linkage maps in melon were constructed using an F_2 _and a RIL population, which were subsequently merged to form a consensus map. Then the scaffolds of the Gy14 and 9930 cucumber draft genomes were associated to markers on both the melon consensus map and a high-resolution cucumber genetic map [43] to define species chromosomes and allow for the detection of syntenic blocks.

## Results

### 1. Development of an F_2_-based extended melon linkage map using cucumber SSRs

In total, 2,487 SSR markers were used to screen for polymorphisms between the two parental lines, Q3-2-2 and Top Mark of the F_2 _mapping population. Of these, 2,442 were genomic SSRs developed from cucumber, 21 from watermelon, 14 from melon [28,29,31], and 10 were EST-SSRs from other species [29]. Of the 2,442 cucumber SSRs, 1,123 (45.9%) produced amplicons in melon after PCR, from which 187 (16.7%) were polymorphic between Top Mark and Q3-2-2. The success in marker transferability for all cucumber SSRs tested was 7.7% (187/2,442). Of the 21 watermelon genomic SSRs that produced amplicons in cucumber (data not presented), seven (33.3%) produced amplicons in melon, and two were polymorphic (2/21 = 9.5%). In total, 196 non-melon polymorphic markers were identified (187 from cucumber, 2 from watermelon, and 7 from other species) and 154 of them were finally placed on the F_2 _genetic map (145 from cucumber, 2 from watermelon and 7 from other species).

Using this F_2 _mapping population, Cuevas et al. [35] mapped 169 co-dominant markers (154 SSR, 8 CAPS, and 7 SNP) in 13 linkage groups (LG). Genotypic data from the 154 additional cucumber or watermelon markers developed herein were combined with the previously mapped 169 markers for linkage analysis. All 323 codominant markers (308 SSRs, 15 CAPS/SNP) were placed onto this F_2_-based melon genetic map in 13 LG. Information on LG and map positions is presented in Table S1 (Additional File 1), and their associated statistics are presented in Table 1. Segregation distortion was rare, where only four markers, CU2186, GCM548, SSR05695, and UW085218 significantly deviated from expected 1:2:1 segregation ratio (*P *< 0.01). Except for Chromosome IV, each LG could be assigned to corresponding chromosomes as defined by Liu et al. [74]. In our analysis, Chromosome IV was split into two linkage groups (4A and 4B) (Table S1) due to an insufficient number of markers. The F_2_-based map consisted of 13 LG spanning 1,012 cM, with a mean marker interval of 3.1 cM, where the largest gap resided in Chromosome IV associated with LG 4A and LG 4B.

**Table 1:** Summary of linkage mapping results for melon (*Cucumis melo *L.) F_2 _(Top Mark × Q3-2-2) and RIL (Top Mark × WI 846-1) mapping populations and a consensus map from F_2 _and RIL map merging

	**F**_**2 **_**Map**^**b**^				**RIL Map**^**b**^				**Consensus Map**^**b**^				**Relative length**^**c**^
**LG (Chr)**^**a**^	# loci mapped	# CS markers	Total cM	Map interval	# loci mapped	# CS markers	total cM	Map interval	# loci mapped	# CS markers	Total cM	Map interval	
1 (I)	31	14	108.7	3.5	32	6	65.1	2.0	38	17	107.3	2.8	9.55
2 (II)	25	14	104.7	4.2	25	9	46.2	1.8	33	19	105.7	3.2	7.50
3 (III)	22	11	81.7	3.7	14	6	46.0	3.3	24	13	81.8	3.4	8.01
4 (IV)	34	16	82.3	2.4	44	15	159.1	3.6	50	26	116.9	2.3	12.61
5 (V)	24	14	87.5	3.6	19	8	85.3	4.5	28	16	58.2	2.1	8.30
6 (VI)	23	10	63.8	2.8	44	9	73.0	1.7	25	11	64.4	2.6	9.25
7 (VII)	28	12	91.9	3.3	37	15	106.5	2.9	41	21	108.0	2.6	7.50
8 (VIII)	35	16	87.1	2.5	21	4	48.3	2.3	39	17	87.2	2.2	7.25
9 (IX)	24	13	71.0	3.0	16	4	42.7	2.7	27	13	71.9	2.7	6.70
10 (X)	20	11	54.2	2.7	23	9	57.4	2.5	30	17	62.5	2.1	9.41
11 (XI)	36	18	100.7	2.8	37	11	89.7	2.4	43	23	87.4	2.0	6.70
12 (XII)	21	7	78.0	3.7	23	3	59.8	2.6	23	6	77.6	3.4	7.21

**Sum/average**	**323**	**156**	**1011.6**	**3.1**	**335**	**99**	**879.1**	**2.6**	**401**	**199**	**1029.0**	**2.6**	**n/a**

^a ^LG = linkage group. Chr = chromosome

^b ^# CS markers = number of markers from cucumber that were mapped in each linkage group.

^c ^Relative length = length of individual chromosome/total length of all 12 chromosomes × 100 [74]

### 2. Development of a RIL-based extended melon linkage map with cucumber SSRs

The molecular markers employed for F_2 _mapping were also used to identify polymorphisms between Top Mark and WI 846-1 of the RIL population. Of the 2,403 cucumber SSRs tested, 44.9% (1,080) produced amplicons in melon, where 11.1% (120/1080) of the SSRs detected polymorphisms between Top Mark and WI 846-1 (5.9% when all 2,043 cucumber SSRs were considered). Of the 127 polymorphic markers identified herein (i.e., 120 from cucumber, 6 from melon or Arabidopsis, and 1 from watermelon), 89 were placed on the RIL map.

Using the same melon RIL population, Cuevas et al. [34] mapped 256 marker loci (105 SSR, 11 SNP/CAPS, and 140 AFLP or RAPDs). The two sets of marker data were pooled for linkage analysis using 80 RILs. Ten of these markers could not be assigned to any LG. The resulting linkage map possessed 335 marker loci including 203 codominant (SSR, CAPS, and SNP) and 132 dominant markers (103 RAPDs and 29 AFLPs) in 22 LG with cumulative genetic distances of 879.1 cM. The mapping data are presented in Table S2 (Additional File 1) and associated map statistics are shown in Table 1. Based on SSR markers shared in common with previously published melon maps [29,34,35,76], it was possible to relegate the LGs constructed herein to 12 linkage groups (chromosomes).

### 3. Development of a consensus melon genetic map through map merging

**Figure 1:**
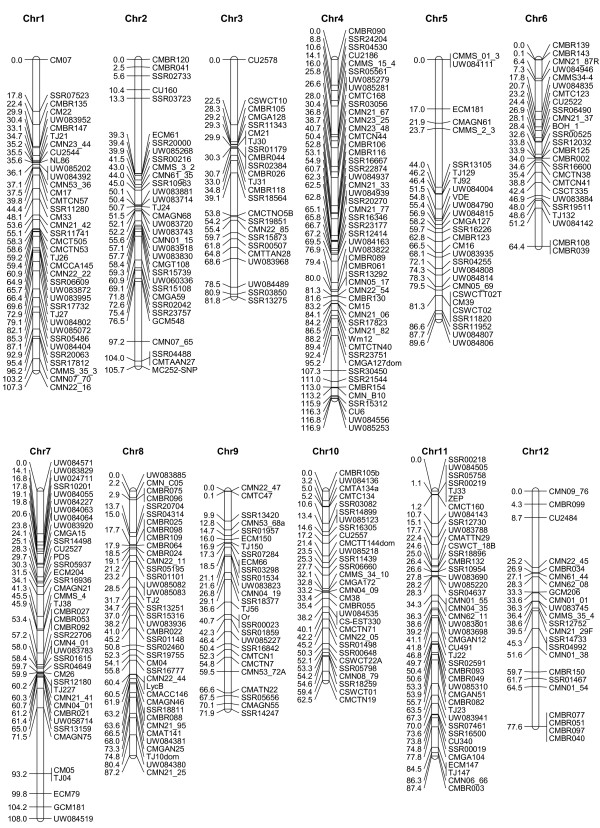
**Consensus linkage map of melon**. This map was developed with the JoinMap 3.0 program by merging the F_2 _and the RIL genetic maps constructed in the present study. The source of each marker and their association with cucumber draft genome scaffolds is provided in Table S3 (Additional File 1). Chr = Chromosome.

The F_2 _and RIL maps developed herein shared 79 markers in common (44 melon, 34 cucumber, and 1 watermelon), and were merged to produce a consensus map using the JoinMap 3.0 program. The 132 dominant AFLP and RAPD markers resident on the RIL map were excluded from map integration because they were deemed ineffective for defining cucumber-melon synteny. These markers tended to clustered on the linkage map, and a number of them showed segregation distortion (data not shown). In contrast to the RIL map, the 323 markers on the F_2 _map were co-dominant and their order was used as the reference during map merging. The resulting consensus linkage map is presented in Figure 1 and summarized in Table 1. Shared loci (boldface typed) between the F_2 _and RIL maps are given in Table S3 (Additional File 1).

The consensus melon genetic map consisted of 401 co-dominant marker loci positioned in 12 LGs spanning 1,029.0 cM with an average marker interval of 2.6 cM. Among the 12 LGs, LG4 (Chromosome IV) had the longest map length (116.9 cM) followed by LG7 (108.0 cM) and LG1 (107.3 cM). In contrast, the length of LG 10 (Chromosome X) was the shortest (62.5 cM). Predictably LG4 possessed the most loci mapped (56) and LG3 (24) the least markers (Table 1). Since marker order of the F_2 _map was used as the reference during map merging, all loci on the consensus map were co-linear with those on the F_2 _map. Moreover, inconsistencies in marker order were rare resulting in substantial colinearity between the RIL map (Table S2) and the consensus map (Table S3, Figure 1).

### 4. Identification of syntenic blocks between melon and cucumber chromosomes

Among the 401 markers present on the consensus melon map, 199 were SSR or CAPS markers developed from cucumber, one from the watermelon, and the remaining 201 were derived from melon. The cucumber Gy14 and 9930 draft genome scaffold locations of these 401 markers were predicted by *in silico *PCR or BLAST searches of the cucumber draft genome sequences.

Seventy-four of the 401 markers on the consensus melon map had no *in silico *PCR products or BLAST hits suggesting that they may be specific to the melon genome. Three markers (CMN53_36, UW084111, and SSR05758) were located in sequences that were annotated as repeated sequences [42], and, therefore, their cucumber scaffold locations were difficult to determine. The remaining 324 markers could be assigned to the Gy14 and 9930 cucumber scaffolds. The scaffold names and physical positions of these markers are presented in Table S3 (Additional File 1).

Once the association of these markers on the melon consensus map with cucumber scaffolds was established, the locations of these cucumber scaffolds in the seven chromosomes of cucumber were deduced from published cucumber genetic maps [43,67-69] (provided in Table S4, Additional File 1). Among the 401 markers placed on the melon consensus map, 79 have been mapped in previous cucumber mapping studies [43,67-69]. Therefore, their locations on cucumber genetic maps could be assigned directly. For markers with associated cucumber scaffold(s), their map locations in the cucumber genome was inferred by other markers derived from the same scaffold, which had been previously placed on cucumber maps (Table S4). However, three markers (UW060336, LycB, and CMCTN7) were located in cucumber scaffolds from which no marker has been mapped. Therefore, their locations in the cucumber genome were unknown.

**Figure 2:**
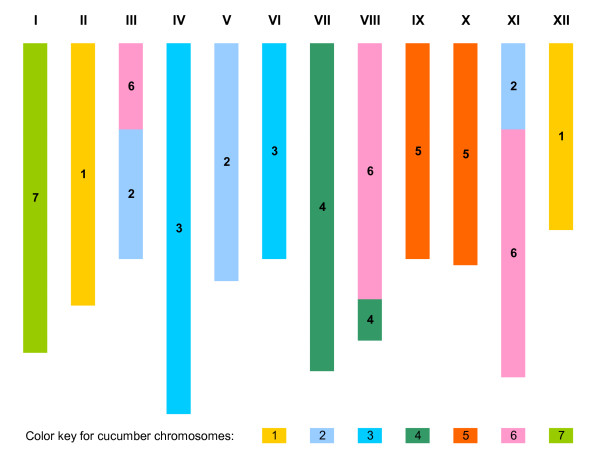
**A cucumber syntenic block view of 12 melon chromosomes (I to XII)**. Number(s) within each melon chromosome indicated corresponding cucumber chromosome numbers with significant synteny. Segments with the same color belong to the same cucumber chromosome. Length of chromosome was drawn based on number of marker loci used for inference of syntenic blocks which is an approximation of actual length of each chromosome.

With molecular markers shared between the melon and cucumber genetic maps or linked by cucumber draft genome scaffolds, syntenic relationships between cucumber and melon chromosomes could be directly inferred. Figure 2 depicts a view of the 12 melon chromosomes consisting of cucumber syntenic blocks (Chromosomes I to XII, where each melon chromosome is portrayed in increasing order of map saturation). Inspection of Figure 2 and Table S3 indicated that melon Chromosome I was syntenic to cucumber Chromosome 7. Likewise, Chromosomes II and XII were syntenic with cucumber Chromosome 1; Chromosomes IV and VI were syntenic with cucumber Chromosome 3; and Chromosomes IX and X were syntenic with cucumber Chromosome 5. Similarly, the three melon chromosomes III, VIII, and XI contained blocks that were syntenic to two cucumber chromosomes, 2+6, 4+6, and 2+6, respectively. These melon-cucumber syntenic relationships are summarized in Table 2.

**Table 2:** Summary of syntenic relationships between cucumber (*Cucumis sativus *L.) and melon (*C. melo *L.) chromosomes

	Corresponding syntenic blocks of melon chromosomes		
**Cucumber Chromosomes**	This study	Huang et al. 2010 [42]	Fukino et al. 2010 [44]

1	II, XII	II, XII	II, XII
2	III, V, XI	III, V	III, V
3	IV, VI	IV, VI	IV, VI
4	VII, VIII	VII	VIII
5	IX, X	IX, X	IX, X
6	III, VIII, XI	III, VIII, XI	III, VIII, XII
7	I	I	I

**Melon chromosomes**	**Corresponding syntenic blocks of cucumber chromosomes**		

I	7		
II	1		
III	2, 6		
IV	3		
V	2		
VI	3		
VII	4		
VIII	4, 6		
IX	5		
X	5		
XI	2, 6		
XII	1		

**Figure 3:**
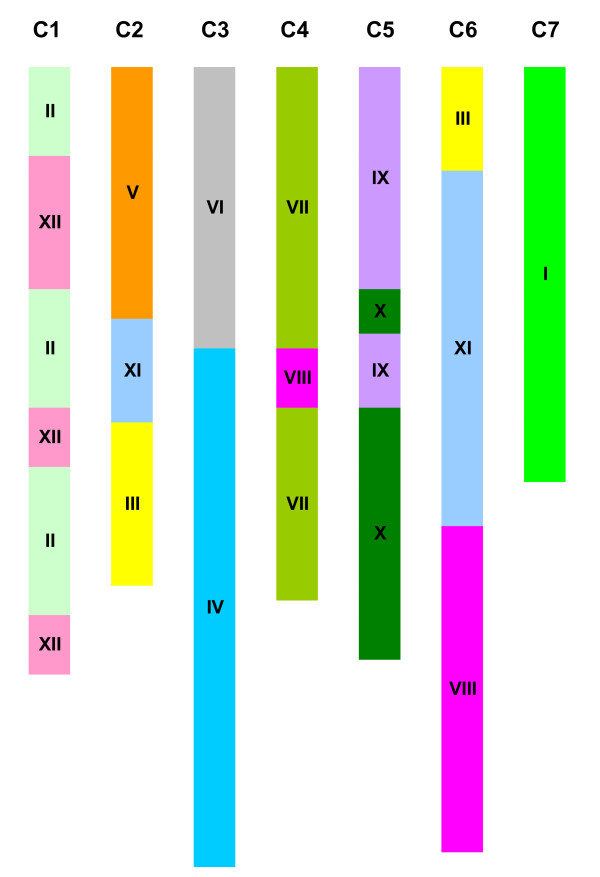
**A melon syntenic block view of 7 cucumber chromosomes (C1 to C7)**. Number(s) within each cucumber chromosome indicated corresponding melon chromosome numbers with significant synteny. Segments with the same color belong to the same melon chromosome. Length of chromosome was drawn based on number of marker loci used for inference of syntenic blocks which is an approximation of actual length of each chromosome.

It has been previously hypothesized that cucumber chromosomes evolved from a progenitor species with 2n = 2 × = 24 chromosomes through chromosome fusion ([42]; also see Discussion below). Thus, a melon syntenic block view of cucumber chromosomes was developed which is shown in Figure 3. In this view, the scaffolds in each cucumber chromosome were arranged in the same order as were marker loci on cucumber genetic maps. In most cases, the high resolution genetic map developed by Ren et al. [43] was used as a reference for ordering those scaffolds. In rare instances, however, marker locations did not coincide with those of Ren et al. [43] and, thus, other more recent cucumber genetic maps [67-69] were consulted to infer marker map locations (data not shown).

The arrangement of melon syntenic blocks across the seven cucumber chromosomes indicates that cucumber chromosome evolution is more complex than simple chromosome fusion (Figure 3). For instance, cucumber Chromosome 7 was homoeologous to melon Chromosome I along its entire length. Cucumber Chromosomes 2 and 6 each contained three syntenic blocks detected in melon Chromosomes V+XI+III, and III+XI+XIII, respectively, and the remaining four cucumber chromosomes (1, 3, 4, and 5) were syntenic with two melon chromosomes but differed in patterns of arrangement of melon syntenic blocks. Cucumber Chromosome 1 was syntenic to melon Chromosome II and XII, whereas cucumber Chromosome 5 was syntenic to melon Chromosome IX and X. In both cases, the syntenic blocks from the two melon chromosomes were arranged alternatively along each cucumber chromosome. In contrast, the syntenic blocks residing in melon Chromosomes VI and IV were in a side-by-side alignment in cucumber Chromosome 3. Lastly, cucumber Chromosome 4 housed syntenic blocks of melon Chromosome VII and VIII, but the syntenic block of melon Chromosome VIII was found to be incorporated into the syntenic block of melon Chromosome VII. Taken collectively, these syntenic patterns were suggestive of a complex history of chromosomal structure changes during cucumber evolution.

### 5. Verification of melon-cucumber syntenic relationships

In this study, *in silico *PCR and BLAST sequence alignment proved useful for inferring the cucumber scaffold location of markers on the melon consensus map (Figures 2 and 3). To verify the syntenic relationships between cucumber and melon chromosomes detected from comparative mapping conducted herein, a similar strategy was used with molecular markers from a melon linkage map developed by Deleu et al. [36]. This gene-based melon linkage map consists of 414 marker loci, of which nearly 200 were SNPs developed from melon EST sequences, and all these markers were derived exclusively from the melon genome. The details of this map are shown in Table S5 (Additional File 1). The genomic or EST sequences from which these markers were developed were used in BLAST analysis against the cucumber draft genome assemblies. Of the 414 markers, only 3 (0.7%) did not yield either *in silico *PCR products or BLAST hits in the cucumber draft genomes examined. This contrasted with markers on the consensus melon map (Table S3), where 81 (20.2%) of 401 (mostly genomic DNA-derived markers) did not produce hits in either Gy14 or 9930 genome assembly. This suggests that the EST sequences examined are highly conserved between these genomes.

The syntenic relationships between melon and cucumber chromosomes inferred from this gene-based melon genetic map are shown in Table S5 (Additional File 1). Although there were no cucumber source markers on this map, melon-cucumber syntenic relationships revealed from the present study (Figures 2 and 3) were confirmed by this independent study [36].

## Discussion

### Cross-species transferability and polymorphisms of cucumber SSRs in the melon genome

Nearly 2,400 randomly sampled cucumber genomic SSRs were tested herein for their ability to detect polymorphisms among three melon parental lines used in map construction. The polymorphism level of cross-species transferable cucumber SSRs was 11.1% between Top Mark and WI 846-1, and 16.9% between Top Mark and line Q3-2-2, which would be reduced to 5.9 and 7.7%, respectively if all cucumber SSRs tested were considered. This level was appreciably lower than that reported in other studies [35,37] using melon SSRs. For example, of 492 melon SSRs evaluated for map construction by Cuevas et al. [35], 32% were polymorphic between two melon lines Q3-2-2 and Top Mark. Likewise, the level of polymorphism of melon SSRs between the two melon lines PI 414723 and Dulce used by Harel-Beja et al. [37] was comparatively high (as much as 55%).

Such differences in polymorphism level may be attributable to the sources of markers (melon source versus cucumber source), the melon lines used, or the relative genetic distance among the germplasm being evaluated. Two additional factors may also contribute to the low polymorphism level detected by cucumber SSRs. Firstly, unlike melon, which is considered genetically diverse [2,3], cucumber has a narrow genetic base [3-6], and the degree of polymorphism at the whole genome level for randomly chosen SSRs between any two cultivated cucumber genotypes is in general less than 20% [69]. In the present study, about 45% of cucumber SSRs examined exhibited cross-species amplification in the melon genome after PCR. This amplification level is similar to that detected by Ritschel et al. [28] who found that ~50% of the melon SSR markers evaluated produced amplicons when cucumber DNAs were used as templates. González et al. [40] found 54% of the melon BAC-end sequences under investigation could be aligned to the cucumber genome. It is likely that the melon genomic regions that were amplified by cucumber SSRs represent conserved portions between the cucumber and melon genomes. The genomes of Gy14 and 9930 cucumber lines have been sequenced using the "next generation" sequencing technologies (Roche 454 and Illumina GAII) [42,66], and, therefore, most of the assembled genome sequences may originate from the gene-rich low or single copy DNA fraction in the genome, which are more conserved between closely related species such as melon and cucumber than repetitive DNA sequences [40,41]. Therefore, using SSRs developed from highly conserved gene-rich genomic DNA regions of a genome with limited genetic diversity may be an important contributor to the low polymorphism of cucumber SSRs in the melon genome. The second factor, as discussed in Cavagnaro et al. [66], is that melon SSRs used in previous mapping studies (e.g., [35,37]) may over-estimate the polymorphism in the genome due to biases created during SSR selection, because of the reiterative selection for longer repeats during development of microsatellite markers. That is, library screening methods for SSR isolation are designed to yield a higher proportion of long SSRs, which are preferentially used for designing primers, which are then typically pre-screened for their relative polymorphism level in potential parental lines. Thus, the most polymorphic loci are selected for further utilization (because there is a positive relationship between SSR length and polymorphism [66,77,78]). In this study, the polymorphism level detected between Q3-2-2 and Top Mark (7.7%) or Top Mark and WI 846-1 (5.9%), although very low, may reflect the true global polymorphism of cucumber SSRs at the whole melon genome level.

### The melon consensus linkage map with cucumber SSRs

The consensus map developed herein added a considerable number of SSR markers (~2 times) towards map saturation in melon (1 < cM between markers). We added 154 and 127 new markers to the previously developed melon F_2 _(from Q-3-2-2 × Top Mark) and RIL (from Top Mark × WI 846-1) maps [34,35], respectively. Marker order on the new (Table S1) and the historic F_2 _maps were almost identical. The RIL map was improved over the historic map [34] (i.e., from 28 LG to 22 LGs), but was still relatively fragmented (i.e., small linkage groups), which was likely due to the large number of dominant markers incorporated into the RIL-based map. It is known that dominant markers such as RAPDs or AFLPs tend to be clustered on the linkage maps [37,67,79-81], and for this reason, dominant markers were excluded in map merging for development of the consensus map.

Even though more than the predicted numbers of LGs were present on the F_2 _and RIL maps, the large number of shared marker loci enabled integration of the two maps to produce a consensus melon genetic map with the expected 12 LGs (Table 1, Figure 1). This consensus map contained 401 co-dominant markers including 199 derived from cucumber, and is the first melon map containing such a high number of cucumber-derived markers. The map length of this consensus map was 1,029 cM, which is within the predicted size range of the melon genome (1,000-1,500 cM) [35]. The reliability (quality) of this map can be measured by its colinearity and its relationship with available cucumber scaffolds. Although there were minor discrepancies in marker orders (Tables S1-S3) between markers on the consensus and the RIL-based maps, these maps were largely collinear (Tables S1-S3). The disparities detected between these maps may be, in part, due to varying recombination rates between mapping population (e.g., RIL vs. F_2_), relatively small mapping populations (i.e., 80 RIL and 91 F_2 _individuals) used, and/or genome structural variations (deletion, inversion, or translocation) between populations. With few exceptions, closely linked markers on these melon maps were located in the same cucumber draft genome scaffolds (Table S3).

### Synteny between melon and cucumber chromosomes

Comparative mapping, *in silico *PCR, and BLAST sequence alignment allowed a comprehensive assessment of syntenic relationships between the cucumber and melon chromosomes (Figures 2 and 3). Among the 401 markers positioned on the melon consensus map, 81 provided no *in silico *PCR products or BLAST hits in the cucumber genome sequences examined (Table S3). Most of these 81 markers were derived from melon genomic DNA sequences (Table S3), indicating their uniqueness to the melon genome. The genomic distribution of these "no-hit" markers also appears to be non-random on the melon consensus map, and in many cases (Chromosomes II, V, VI, VIII, IX, X, XI, and XII), these markers were located at telomeric ends, implying the possibility of rapid evolutionary divergence (Table S3). Such information may allow design of experiments to investigate the nature of these regions in *Cucumis *chromosome evolution.

Phylogenetic analyses [8,10,12] in *Cucumis *support an early hypothesis that the base chromosome number *x *= 7 in cucumber is derived from a progenitor species with *x *= 12 [17,18]. Six of the seven cucumber chromosomes may be originated from chromosome fusion of such a progenitor species [42,44]. We provide herein additional information regarding the syntenic relationships between cucumber and melon chromosomes indicating that the evolutionary dynamics of cucumber evolution is complex (Table 2, Figure 3). Although we confirmed the early finding [42,44] that cucumber Chromosome 7 was syntenic with melon Chromosome I, we found that the co-linearity of marker loci in the two chromosomes has been broken (Table S3, Additional File 1) suggesting that Chromosome 7 of cultivated cucumber may have undergone certain structural changes since its divergence from an ancestral species (e.g., melon or *C. hystrix*, see below). The other six chromosomes of cucumber appear to have more complicated evolutionary histories. For instance, both Chromosomes 2 and 6 possess genomic regions that are syntenic to genetic blocks from at least three melon chromosomes (III+V+XI, and III+VIII+XI, respectively; Figure 3), suggesting they are derived from more than simple chromosome fusion. Although Chromosomes 1, 3, 4, and 5 each possessed genomic blocks syntenic with those from two melon chromosomes, the arrangements of these blocks differed among chromosomes (Figure 3). Since cucumber Chromosome 3 (longest of the 7 chromosomes) [70,72] appears to be comparatively simple in its syntenic block organization, it is tempting to hypothesize that it originated from simple fusion of melon chromosomes IV and VI. However, close inspection of marker order in cucumber Chromosome 3 and melon Chromosomes IV and VI (Table S3) reveals non-colinearity, which suggests that additional structural changes may have occurred after a hypothetical chromosome fusion event.

Data presented herein provides a rather complicated description of *Cucumis *species chromosome evolution, making a comprehensive statement regarding chromosome evolution in melon and cucumber difficult at this time. It is apparent that additional information is required regarding the genomic nature of melon, and cucumber, as well as potential 'bridge" species [[12], e.g., *C. hystrix*) to elucidate more clear evolutionary relationships in this genus. To better understand these relationships a more saturated cucumber genetic map is needed to increase the resolution of cucumber scaffolds for comparative mapping. The cucumber map used herein was developed with 77 RILs from an inter-subspecific cross between cultivated cucumber (*C. sativus *var. *sativus*) line Gy14 and the wild *C. sativus *var. *hardwickii *line PI 183967 [43]. There were, however, strong recombination suppressions detected during map construction resulting in clustering of molecular markers in several regions of cucumber Chromosomes 4, 5, and 7. Such recombination suppression may, in part, be due to structural differences in chromosomes between cultivated and wild cucumbers (Table S3), which, in turn, may have reduced the power of ordering markers and scaffolds. Meanwhile, the whole genome sequencing of melon is near completion (Garcia-Mas et al., manuscript in preparation). This will provide a powerful tool to understand the synteny between the melon and cucumber chromosomes at DNA sequence level.

A wild relative of cucumber, *C. hystrix *(2n = 2 × = 24), may play the key role in understanding the process of cucumber chromosome evolution. Extensive phylogenetic analyses in *Cucumis *have indicated that *C. hystrix *is so far the closest wild relative of cucumber [8,10,12]. The lack of genetic affinity between *C. hystrix *and *C. melo*, and African species, lends support to the hypothesis that *C. hystrix *is a progenitor species of *C. sativus*, or that they at least share a common ancestral lineage [82]. *C. hystrix *has the same number of chromosomes as melon, and is sparingly cross-compatible with cucumber, but not melon [83]. A fertile amphidiploid, *C. hystivus *(2n = 4 × = 38) between cucumber and *C. hystrix*, as well as alien addition lines have also been successfully created [84,85]. The seven meiotic chromosomes of *C. sativus *are larger than the 12 chromosomes of *C. hystrix *[85]. Fluorescence *in situ *hybridization of 45S rDNA and CsCent1 repetitve DNA probes to cucumber pachytene chromosomes also suggested that cucumber Chromosomes 1 and 2 may have evolved from fusions of an ancestral karyotype with 2n = 24 [86]. These findings make *C. hystrix *the primary resource for testing genetic hypotheses for further characterizing the evolution of modern cucumber.

## Conclusions

This is the first broad-based comparative analysis of synteny between melon and cucumber. The consensus melon linkage map derived herein from two historic maps possesses the largest number of cross-species cucumber molecular markers currently mapped in the melon genome and provides a greater understanding of genomic relationships between these two important *Cucumis *species. Data support the hypothesis that cucumber chromosomes originate from fusions of chromosomes of an *x *= 12 ancestral progenitor. However, many structural changes may have occurred in the evolution of the seven cucumber chromosomes. In depth cytogenetic and molecular investigations of *x *= 12 *Cucumis *species (e.g., melon and *C. hystrix*) will likely provide further evidence of chromosome evolution in cucumber, melon and related taxons.

## Methods

### Plant materials

Two melon populations were employed to conduct linkage mapping experiments using cucumber microsatellite markers. The first experiment used 91 F_2 _plants derived from a cross between melon inbred lines Top Mark and Q3-2-2. Top Mark is an andromonoecious U.S. Western Shipping market class melon in Group Cantalupensis [87]. The monoecious line Q3-2-2 does not fall into any common market class, but possesses Group Conomon and Momordica melon morphological fruit characteristics [88]. The second experiment used 80 F_8 _RIL derived from Top Mark × WI 846-1 [34]. The monoecious line WI 846-1 was derived through selfing from a three-way cross between a *C. melo *ssp. *agrestis *(Naud.) Pangalo germplasm (Cartago, Costa Rica), an Eastern market type breeding line (USDA, ARS, Clemson SC), and a Galia market type breeding line (ARO, Israel) [34]. Both populations were previously used in the mapping of fruit yield- and quality-related components in melon [34,35,89,90].

### Molecular marker analysis

Two sets of SSR markers developed from cucumber whole genome sequences were employed for polymorphism screening and the development of melon linkage maps. The first set included 2,012 SSR markers from the draft genome of cucumber inbred line 9930 [43] and the second set included a collection of 83,689 SSRs developed from the Gy14-derived draft genome [66]. Also employed were 21 watermelon microsatellite markers known to amplify in the cucumber genome (U. Reddy, unpublished data). Information regarding all mapped markers used herein (i.e., primer sequences and their scaffold locations in the Gy14 and 9930 draft genomes) is provided in Table S3 (Additional File 1).

For DNA extraction, unexpanded young leaves from each F_2 _plant or five plants from each RIL were placed into a 2.0 ml microcentrifuge tube, lyophilized in a freeze dryer, and ground into fine powder in a high-throughput homogenizer (OPS Diagnostics, Lebanon, NJ, USA). Genomic DNA from all samples was extracted using the CTAB method [91].

Each polymerase chain reaction (PCR) contained 25 ng template DNA, 0.5 μM each of forward and reverse primers, 0.2 mM dNTP mix, 0.5 unit of *Taq *DNA polymerase and 1× PCR buffer (Fermentas, Glen Burnie, MD, USA) in a total volume of 10.0 μl. A "touch-down" PCR program was employed for all primer sets [92]. The PCR products were size-fractionated in a 9% polyacrylamide gel. After gel electrophoresis, band patterns were visualized with silver staining, and gel images were taken with a digital camera.

### Linkage map construction and map integration

Previously, 169 and 256 markers loci were positioned, respectively, on Q3-2-2 × Top Mark F_2 _and WI 846-1× Top Mark melon RIL linkage maps [34,35]. Genotypic marker data from previous mapping studies and the present study in each population were combined for linkage analysis. For each marker, Chi-square analysis was performed to test goodness-of-fit to expected 1:2:1 segregation ratio for the F_2 _population and 1:1 ratio for the RIL population (significance declared at *P *< 0.01). Linkage analysis was then performed with JoinMap 3.0 with three rounds of mapping to build the map, and LG were established at a LOD threshold of 4.0 with the Kosambi function.

Seventy-nine markers were shared in common between the F_2 _and the RIL maps. A consensus melon map was developed by merging the two maps according to Cuevas et al. [35]. Common markers having the same recombination fraction (rf) among different populations are required for use as anchor makers during map merging [93]. Therefore, heterogeneity test was conducted for these common markers using JoinMap algorithms to determine recombination fractions in both populations (RIL and F_2_). Common markers with different recombination fraction (*P *< 0.05) were excluded from map merging experiments. All AFLP and RAPD markers present on the RIL-based map were also excluded from map integration given their inability to provide appropriate information for assessment of syntenic relationships. Subsequently, these maps (F_2 _and RIL) were merged using JoinMap 3.0 (LOD = 2.0; rf = 0.35-0.50) by employing the "*fixed order*" option according to Qi et al. [94] using the marker order present on the F_2 _map as the reference. This option allowed for the definition of fixed order marker subsets based on marker order in the F_2 _linkage groups.

The numbering conventions for melon linkage groups and their respective chromosome assignment followed Liu et al. [74], which is consistent with the nomenclature established by the melon research community ([76], also see http://www.icugi.org/). To avoid confusion, cucumber chromosomes were named using Arabic numbers (1 through 7), whereas the Roman numerals I to XII were used to indicate melon Chromosomes 1 to 12, respectively.

### Inference of syntenic relationships between the melon and cucumber genomes

The melon consensus linkage map (401 marker loci) was used to infer syntenic relationships between melon and cucumber chromosomes. The map/chromosomal locations of these markers in the cucumber genome were inferred from procedures given below.

Initially, four SSR-based cucumber genetic maps [43,67-69] were used as a primary reference to determine the chromosomal locations of mapped loci including a high-resolution genetic map (with 995 SSR loci) [43]. These maps contained 1,244 unique SSR or SCAR marker loci distributed in seven cucumber chromosomes. The associated scaffold locations of the majority of these markers in the 9930 or Gy14 draft genome were known [66]. The map locations as well as their chromosomal assignment and associated scaffolds in the 9930 and Gy14 draft genomes of the 1,244 mapped loci are summarized in Table S4 (Additional File 1).

Of the 401 markers positioned on the melon consensus map, 199 originated from cucumber, of which 79 were present on different cucumber genetic maps [43,67-69]. Therefore, the cucumber scaffolds associated with these 79 markers were known. For other markers, an *in silico *PCR (virtual PCR) strategy [66] was used to deduce their position on the cucumber scaffold of the Gy14 and 9930 draft genome assemblies. For each marker, the output of *in silico *PCR included the scaffold name, nucleotide positions of left and right primer binding sites, and the sequence and expected size of virtual PCR product. The information was generated and recorded for both 9930 and Gy14 draft genomes and is summarized in Table S3 (melon consensus map) and Table S4 (cucumber linkage maps). In cases where no or multiple *in silico *PCR products were available, the marker was labeled 'no hit' or 'multi-copy', respectively (Tables S3-S5).

Many markers mapped on the melon consensus map were developed from EST or gene regions (e.g., EST-SSR, or EST-SNP, or genes). In this case, the source DNA sequences were used to perform a BLAST search against the draft genome to find their (marker) scaffold locations. Most of the sequences examined were extracted from the s (http://www.ncbi.nih.gov/), the International Cucurbit Genome Initiative (http://www.icugi.org/), and the MELOGEN (http://www.melogen.upv.es/) databases. Cuevas et al. [34,35] mapped six melon genes in the carotenoid pathway which included genes for the phytoene synthase (*PS*), beta-carotene hydroxylase-1 (*BOH-1*), lycopene cyclase (*LycB*), zeaxanthin epoxidase (*ZEP*), phytoene desaturase (*PDS*), violaxanthin de-epoxidase (*VDE*), and the orange-related gene (*Or*). These gene sequences were also employed in BLAST search against the cucumber draft genomes to identify the scaffold locations of their homologous sequences.

Once a marker was assigned to the cucumber draft genome scaffold, its map location was inferred in two ways. Firstly, 79 of the 401 markers had already been mapped in cucumber chromosomes, and they could be directly assigned to specific chromosomal location(s) of the cucumber genome. Secondly, if a marker had not been mapped in cucumber but the cucumber scaffold possessed markers that have been mapped in the cucumber genome, then that marker was assigned to the same location as those markers.

### Verification of melon-cucumber syntenic relationships

A melon genetic map developed by Deleu et al. [36] was used to verify the syntenic relationships between melon and cucumber chromosomes identified by this study. It was selected because it contained 414 markers in 12 LGs, of which nearly half were gene-based SNPs. All the 414 markers were developed exclusively from the melon genome. Genomic or EST sequences from which these markers were developed were downloaded from databases located at MELOGEN (http://www.melogen.upv.es/), GenBank (http://www.ncbi.nih.gov/) or at ICUGI (http://www.icugi.org/) websites. The majority of these markers could be mapped onto cucumber scaffolds using both *in silico *PCR and BLAST sequence alignment. All information for molecular markers on this consensus melon genetic map is provided in Table S5 (Additional File 1).

## Abbreviations

AFLP: amplified fragment length polymorphism; BLAST: basic local alignment search tool; CAPS: cleavaged amplified polymorphic sequence; EST: expressed sequence tag; FISH: fluorescence in situ hybridization; Mbp: million base pairs; LG: linkage groups; PCR: polymerase chain reaction; RAPD: random amplified polymorphic DNA; QTL: quantitative trait loci; RIL: recombinant inbred line; SCAR: sequence characterized amplified region; SNP: single nucleotide polymorphism; SSR: simple sequence repeats.

## Authors' contributions

DL performed the majority of the experiments. YW conceived the study, designed the experiments, analyzed the data and wrote the manuscript along with editing and suggestions by JES. HC, JZ, and JES developed the melon populations and initial F_2 _and RIL melon genetic maps used as backbones for the consensus map. LY, YL and XH conducted linkage mapping in cucumber. JG-M developed the EST-based melon linkage map. UR developed watermelon SSRs and conducted polymorphisms screening of melon parental lines. FL screened additional melon SSRs for polymorphism between parental lines. All authors read and approved the final manuscript.

## Supplementary Material

Additional file 1 **supplemental data file including 5 supplemental MS Excel tables (Table S1 to Table S5). Table S1**. Melon (*Cucumis melo *L.) linkage map developed from the Q3-2-2 × Top Mark F_2 _mapping population. LG 1 to 12 corresponded to melon Chromosomes I to XII, respectively. **Table S2**. Linkage map of melon (*Cucumis melo *L.) developed from a RIL mapping population between WI 846-1 and Top Mark. LG 1 to 12 corresponded to melon Chromosomes I to XII, respectively. **Table S3**. The melon (*Cucumis melo *L.) consensus map developed from map integration of the F_2 _(Q3-2-2 × Top Mark) and RIL (WI 846-1 × Top Mark) linkage maps, and syntenic relationships of cucumber (*C. sativus *L.) and melon chromosomes. **Table S4**. Molecular markers mapped in cucumber (*Cucumis sativus *L.) and their associated scaffolds in the Gy14 and 9930 draft genomes. The map positions of these scaffolds on cucumber maps were used to infer syntenic blocks between melon (*C. melo *L.) and cucumber chromosomes. **Table S5**. Cucumber-melon chromosome syntenic relationships inferred from the EST-based melon linkage map by Deleu et al. [36]. Primer sequences of highlighted markers were not presented in Deleu et al. [36] Click here for file
